# Case Report: Surgical management of giant hepatic cavernous haemangioma with Kasabach–Merritt syndrome in an adult

**DOI:** 10.3389/fmed.2026.1896193

**Published:** 2026-07-13

**Authors:** Liuyang Xu, Jianwei Bai, Zhenqian Tu, Yuan Zhu, Zeng Ye, Xiaofeng Liao, Xiaogang Li

**Affiliations:** 1 Department of Biliary and Pancreatic, Xiangyang Central Hospital, School of Medicine, Wuhan University of Science and Technology, Wuhan, China; 2 Department of Biliary and Pancreatic Surgery, Xiangyang Central Hospital, Affiliated Hospital of Hubei University of Arts and Science, Xiangyang, China; 3 Department of Pancreatic Surgery, Fudan University Shanghai Cancer Center, Shanghai, China

**Keywords:** adult, coagulopathy, hepatectomy, hepatic cavernous haemangioma, Kasabach–Merritt syndrome, liver surgery, thrombocytopenia

## Abstract

A 25-year-old woman presented with a progressively enlarging abdominal mass and severe coagulopathy (fibrinogen 0.6 g/L). Imaging revealed a giant, predominantly calcified hepatic lesion lacking typical nodular enhancement, raising suspicion for hamartoma or cystadenoma. Despite aggressive fibrinogen replacement, coagulopathy persisted. Multidisciplinary team discussion confirmed Kasabach-Merritt syndrome secondary to giant hepatic haemangioma. Anatomical left lateral sectionectomy was performed successfully. Histopathology confirmed cavernous haemangioma with thrombosis, infarction, and calcification. Coagulation parameters normalized immediately postoperatively. This case highlights the diagnostic pitfall of non-enhancing giant hepatic haemangioma and underscores the curative role of early surgical resection in adult Kasabach-Merritt syndrome.

## 1 Introduction

Hepatic cavernous haemangioma is the most common benign liver tumor. However, its association with Kasabach-Merritt syndrome (KMS)—a consumptive coagulopathy characterized by thrombocytopenia and hypofibrinogenemia—is exceedingly rare in adults. Hepatic haemangiomas occur in approximately 5%−7% of adults, whereas giant forms (>5 cm) are uncommon (1). KMS complicates < 1% of these giant lesions and has been reported in fewer than 100 adult cases worldwide (2, 4). Without definitive intervention, mortality exceeds 30%, primarily due to refractory coagulopathy and hemorrhagic complications (4). Current literature largely focuses on pediatric vascular tumors, while adult KMS remains under-recognized. Furthermore, giant calcified haemangiomas may exhibit atypical imaging features, mimicking malignancies or cystic lesions and delaying definitive treatment. This case illustrates the diagnostic challenge posed by a non-enhancing giant hepatic haemangioma and demonstrates a successful surgical strategy.

## 2 Case description

A 25-year-old woman presented with a 3-year history of an enlarging abdominal mass. Her medical history included anemia, chronic urticaria, and a prolactinoma managed with bromocriptine. Physical examination revealed a firm, non-tender, 20 × 15 cm mass occupying the epigastrium. Laboratory tests showed hemoglobin 71 g/L, platelets 110 × 10^9^/L, and a critically low fibrinogen level of 0.6 g/L, with elevated D-dimer (20 mg/L). Liver and renal function were otherwise normal (Supplementary Table 1). Baseline laboratory records from 3 months prior were unavailable, as the initial workup was performed at an outside hospital. According to the patient's recall, anemia had been identified 6 months prior but remained untreated.

Contrast-enhanced CT revealed a massive, predominantly calcified mass in the left hepatic lobe. Notably, the lesion exhibited no significant enhancement in the arterial, portal venous, or delayed phases, raising suspicion for a hepatic hamartoma or cystadenoma (Figure 1). MRI demonstrated heterogeneous signal intensity with internal septations but lacked classic peripheral nodular enhancement (Figure 2).

**Figure 1:**
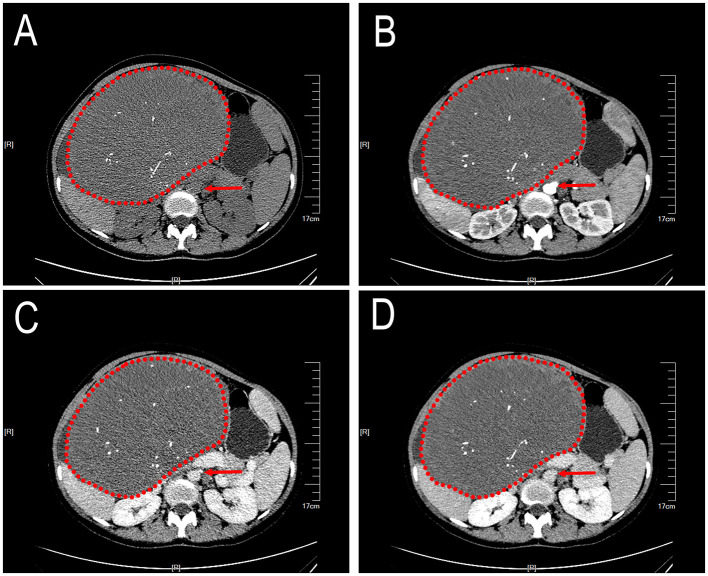
Computed tomography images demonstrating a predominantly calcified hepatic mass (red dotted line) exhibiting no significant enhancement across all phases. **(A)** Non-contrast, **(B)** arterial, **(C)** portal venous, and **(D)** delayed phases. The red arrow points to the abdominal aorta, which shows normal enhancement, highlighting the avascular nature of the mass.

**Figure 2:**
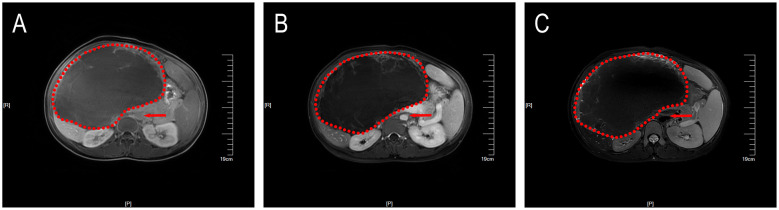
Magnetic resonance imaging demonstrating a giant hepatic haemangioma (red dotted line). **(A)** T1-weighted, **(B)** T2-weighted, and **(C)** contrast-enhanced T1-weighted images show heterogeneous signal intensity and internal septations without typical nodular enhancement. The red arrow points to the abdominal aorta, serving as a reference for vascular enhancement.

Multidisciplinary team (MDT) discussion during this admission initially suspected hepatic hamartoma based on the non-enhancing, calcified appearance on CT (Figure 1). Differentials such as autoimmune hemolysis or bromocriptine-related toxicity were excluded given the chronic DIC-like picture. Severe transfusion reactions necessitated switching to washed red blood cells and fibrinogen concentrate. Despite 3 days of supplementation, fibrinogen remained refractory (0.6 g/L), prompting a second MDT discussion. The team concluded that the giant tumor was consuming fibrinogen faster than replacement and increased the dosage. Following this adjustment, fibrinogen levels improved to 1.52 g/L (Figure 3), meeting the threshold for safe surgery.

**Figure 3:**
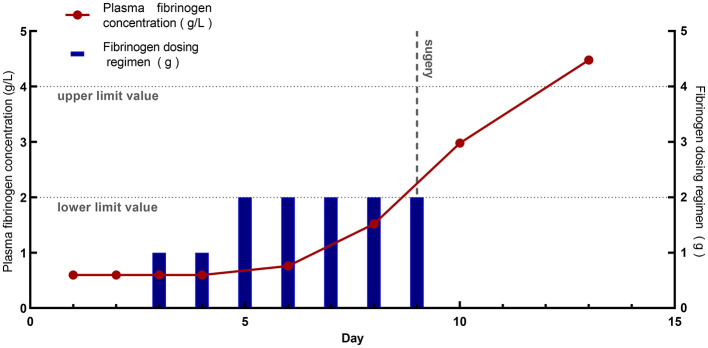
Timeline of fibrinogen levels and transfusion regimen during hospitalization.

On day 9, the patient underwent an exploratory laparotomy. Intraoperative findings confirmed an 18 × 15 × 22 cm mass arising from the left lateral lobe (Supplementary Figure 1). An anatomical left lateral hepatectomy was performed with complete *en bloc* excision and minimal blood loss (100 ml). Histopathological examination confirmed a cavernous haemangioma with extensive thrombosis, infarction, and calcification (Supplementary Figure 2). Postoperatively, the patient required no further transfusions. Fibrinogen levels surged to 2.98 g/L by postoperative day 1, and hemoglobin normalized to 115 g/L by day 3. The patient was discharged on postoperative day 6, and followed up 9 days after discharge. Routine blood tests showed complete normalization of hematological parameters, with hemoglobin restored to 132 g/L and platelets to 151 × 10^9^/L, confirming the resolution of Kasabach-Merritt syndrome following surgical resection.

## 3 Discussion

Hepatic cavernous haemangioma is the most common benign liver tumor; however, its association with KMS is exceedingly rare in adults (1). KMS is characterized by thrombocytopenia and consumptive coagulopathy due to platelet sequestration within the vascular tumor bed (2).

The most striking feature of this case was the atypical imaging. Classic haemangiomas exhibit progressive centripetal enhancement. In contrast, this mass showed no enhancement across all phases. We attribute this to the tumour's massive size, internal calcification, and fibrous replacement, which impeded blood flow into the vascular spaces (3). This led to a preoperative diagnostic dilemma, mimicking a hamartoma or cystadenoma. Clinicians should maintain a high index of suspicion for haemangioma even in the absence of typical enhancement, especially when accompanied by coagulopathy.

The consumptive coagulopathy in KMS is driven by platelet sequestration and activation within the massively dilated sinusoidal spaces of the giant haemangioma, leading to continuous consumption of clotting factors, particularly fibrinogen (2). In lesions exceeding 10 cm, sluggish intratumoural flow promotes intralesional microthrombus formation and traps plasma proteins faster than physiological or standard replacement can compensate (1). This “sponge effect” explains why initial low-dose fibrinogen supplementation remained refractory until the replacement dosage was escalated under MDT guidance.

The second challenge was the refractory coagulopathy. Despite aggressive fibrinogen replacement, levels remained subnormal. This resistance is a hallmark of KMS, reflecting continuous consumption by the tumor (4). While medical therapies like sirolimus exist, their onset is slow (5). Transarterial chemoembolisation (TACE) is generally contraindicated due to the risk of exacerbating coagulopathy (6).

Surgical resection remains the definitive treatment (7). By removing the source of consumption, we observed an immediate and sustained normalization of coagulation profiles. Given the patient's young age, localized disease, and sufficient future liver remnant, anatomical hepatectomy was the safest curative option. This case underscores the necessity of MDT discussions to navigate complex diagnostic and therapeutic decisions.

## 4 Conclusion

This case highlights a giant hepatic haemangioma with atypical non-enhancing appearance and internal calcification, presenting with Kasabach–Merritt syndrome. Complete surgical resection proved curative, rapidly reversing life-threatening coagulopathy and restoring hematological normality. Given residual small haemangiomas, regular imaging surveillance is strongly recommended.

## Data Availability

The original contributions presented in the study are included in the article/Supplementary material, further inquiries can be directed to the corresponding authors.

## Supplementary material

Supplementary Figure 1 Intraoperative photograph of the resected specimen showing the gross appearance of the giant hepatic cavernous haemangioma. The specimen measured 18 × 15 × 22 cm. Note the lobulated contour and extensive whitish calcifications scattered across the surface.

Supplementary Figure 2 Histopathological examination of the resected mass. (A) Overview image showing a large area of infarction (region delineated by the black dashed line). (B) Close-up view revealing multiple variably sized, blood-filled vascular channels lined by a single layer of flattened endothelial cells, confirming the diagnosis of cavernous haemangioma.

Supplementary Table 1 Laboratory parameters at presentation.
