# Supplementary figures and images for: Case Report: Surgical management of giant hepatic cavernous haemangioma with Kasabach–Merritt syndrome in an adult
Source: Front Med (Lausanne). 2026 Jul 13;13:1896193. doi: 10.3389/fmed.2026.1896193 (PMC13402568; doi:10.3389/fmed.2026.1896193)

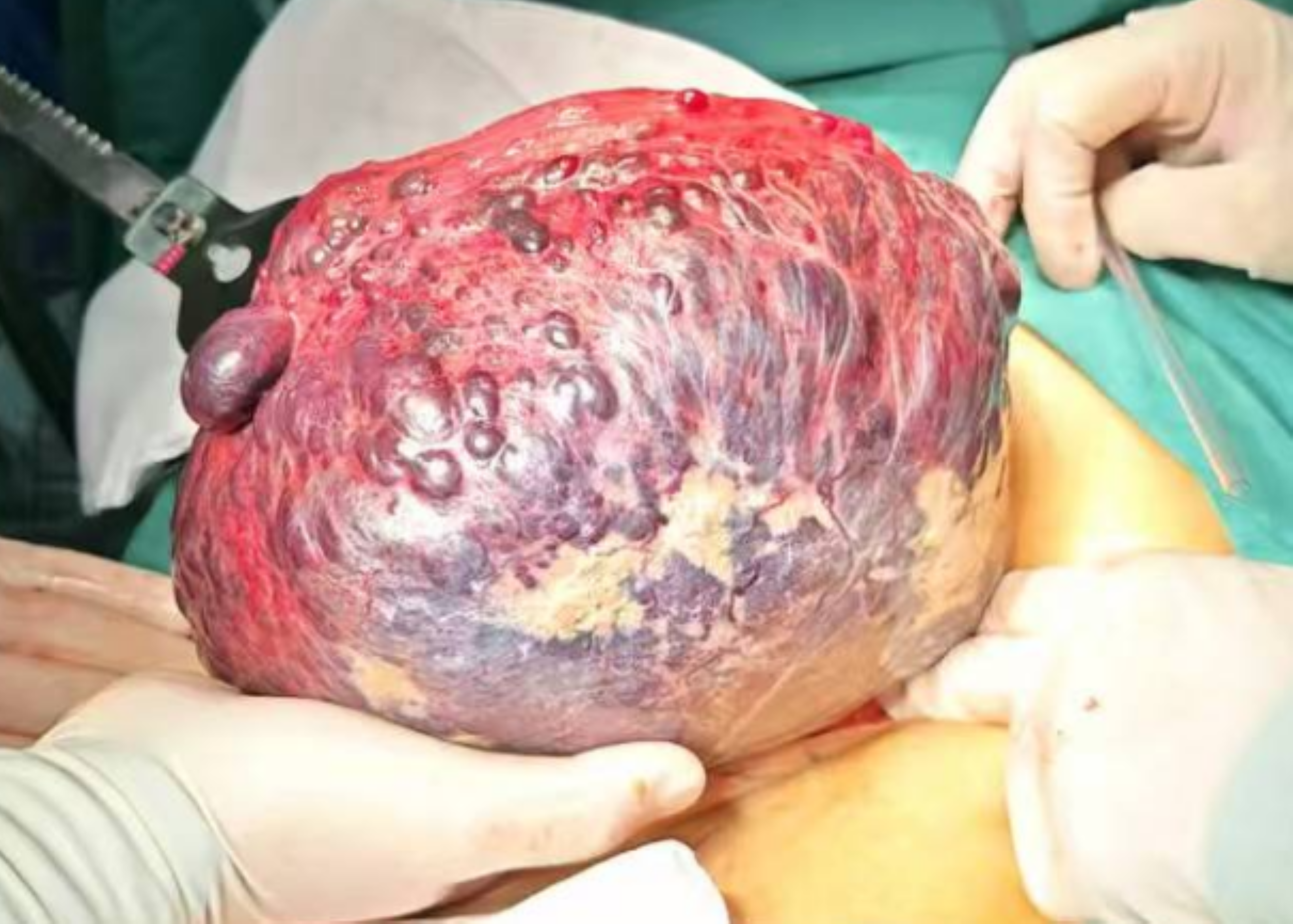

Supplement: Supplementary Figure 1 — Intraoperative photograph of the resected specimen showing the gross appearance of the giant hepatic cavernous haemangioma. The specimen measured 18 × 15 × 22 cm. Note the lobulated contour and extensive whitish calcifications scattered across the surface. [file Image_1.TIF]

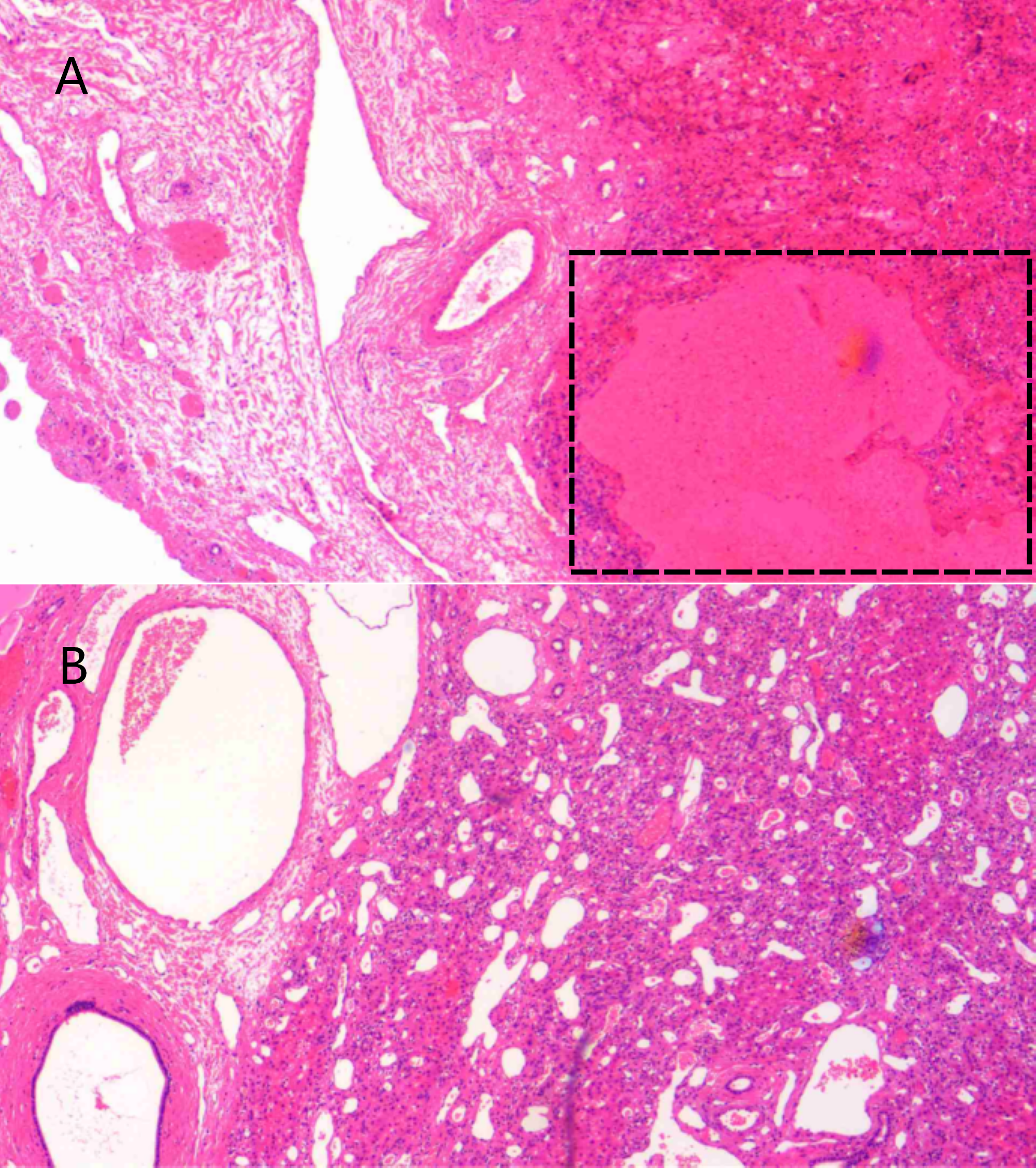

Supplement: Supplementary Figure 2 — Histopathological examination of the resected mass. (A) Overview image showing a large area of infarction (region delineated by the black dashed line). (B) Close-up view revealing multiple variably sized, blood-filled vascular channels lined by a single layer of flattened endothelial cells, confirming the diagnosis of cavernous haemangioma. [file Image_2.TIF]
