# Supplementary material for: Case Report: Surgical management of giant hepatic cavernous haemangioma with Kasabach–Merritt syndrome in an adult
Source: Front Med (Lausanne). 2026 Jul 13;13:1896193. doi: 10.3389/fmed.2026.1896193 (PMC13402568; doi:10.3389/fmed.2026.1896193)
Supplement: Supplementary Table 1 — Laboratory parameters at presentation. [file Table_1.docx]

**Case Report: Surgical management of giant hepatic cavernous haemangioma with Kasabach‑Merritt syndrome in an adult**

sTable 1. Laboratory parameters at presentation.

| Parameter | Patient Value | Reference Range |
| --- | --- | --- |
| Haemoglobin | 71 g/L | 115-150 g/L |
| Platelets | 110 × 10⁹/L | 125-350 × 10⁹/L |
| Fibrinogen | 0.6 g/L | 2.0-4.0 g/L |
| D-dimer | 20 mg/L | < 0.5 mg/L |
| Total Bilirubin  Direct Bilirubin  ALT  AST  Albumin  Creatinine  eGFR | 15.4 μmol/L  5.3 μmol/L  26 U/L  31 U/L  48.6 g/L  45.7 μmol/L  133.48 ml/min/1.73m² | 0.0-23.0 μmol/L  0.0-8.0 μmol/L  7-40 U/L  13-35 U/L  40.0-55.0 g/L  41.0-73.0 μmol/L  >90 ml/min/1.73m² |
