# Supplementary material for: Case Report: Surgical management of giant hepatic cavernous haemangioma with Kasabach–Merritt syndrome in an adult
Source: Front Med (Lausanne). 2026 Jul 13;13:1896193. doi: 10.3389/fmed.2026.1896193 (PMC13402568; doi:10.3389/fmed.2026.1896193)
Supplement: Supplementary file 4 [file Table_2.docx]

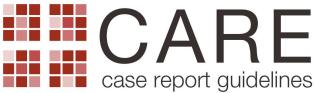
CARE Checklist of information to include when writing a case report
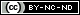


**Topic Item Checklist item description Reported on Line**

**Title 1** The diagnosis or intervention of primary focus followed by the words “case report” . . . . . . . . . . . . . . . . . . Yes – Title

**Key Words 2** 2 to 5 key words that identify diagnoses or interventions in this case report, including "case report" Yes – Keywords

**Abstract**

**(no references)**

**3a** Introduction: What is unique about this case and what does it add to the scientific literature? Yes – Abstract

**3b** Main symptoms and/or important clinical findings . . . . . . . . . . . . . . . . . . . . . . . . . . . . . . . . . . . . . . . . . . . . . . . . . . . Yes – Abstract

**3c** The main diagnoses, therapeutic interventions, and outcomes Yes – Abstract

**3d** Conclusion—What is the main “take-away” lesson(s) from this case? Yes – Abstract

**Introduction 4** One or two paragraphs summarizing why this case is unique (**may include references**) Yes – Introdoction

**Patient Information 5a** De-identified patient specific information Yes – Case Description

**5b** Primary concerns and symptoms of the patient Yes – Case Description

**5c** Medical, family, and psycho-social history including relevant genetic information Yes – Case Description

**5d** Relevant past interventions with outcomes Yes – Case Description

**Clinical Findings**

**Timeline**

**Diagnostic Assessment**

**Therapeutic Intervention**

**Follow-up and Outcomes**

1. Describe significant physical examination (PE) and important clinical findings Yes – Case Description
2. Historical and current information from this episode of care organized as a timeline Yes – Figure 3

**8a** Diagnostic testing (such as PE, laboratory testing, imaging, surveys). Yes – Case Description

**8b** Diagnostic challenges (such as access to testing, financial, or cultural) Yes – Discussion

**8c** Diagnosis (including other diagnoses considered) Yes – Case Description

**8d** Prognosis (such as staging in oncology) where applicable Yes – Discussion

**9a** Types of therapeutic intervention (such as pharmacologic, surgical, preventive, self-care) . . . . . . . . . . . . . . . . . Yes – Case Description

**9b** Administration of therapeutic intervention (such as dosage, strength, duration) Yes – Case Description

**9c** Changes in therapeutic intervention (with rationale) Yes – Case Description

**10a** Clinician and patient-assessed outcomes (if available) Yes – Case Description

**10b** Important follow-up diagnostic and other test results Yes – Case Description

**10c** Intervention adherence and tolerability (How was this assessed?) Yes – Case Description

**10d** Adverse and unanticipated events N/A

**Discussion 11a** A scientific discussion of the strengths AND limitations associated with this case report Yes – Discussion

**11b** Discussion of the relevant medical literature **with references** Yes – Discussion

**11c** The scientific rationale for any conclusions (including assessment of possible causes) Yes – Discussion

**11d** The primary “take-away” lessons of this case report (without references) in a one paragraph conclusion Yes – Discussion

**Patient Perspective 12** The patient should share their perspective in one to two paragraphs on the treatment(s) they received . . . . N/A

**Informed Consent 13** Did the patient give informed consent? Please provide if requested . . . . . . . . . . . . . . . . . . . . . . . . . . . . . . . . . . . . . . **Yes** ☑ **No
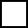
**
